# Effectiveness of Combined Pulmonary Rehabilitation and Progressive Muscle Relaxation in Treating Long-Term COVID-19 Symptoms: A Randomized Controlled Trial

**DOI:** 10.3390/jcm13206237

**Published:** 2024-10-18

**Authors:** Adelina Maritescu, Alexandru Florian Crisan, Camelia Corina Pescaru, Emil Robert Stoicescu, Cristian Oancea, Daniela Iacob

**Affiliations:** 1 Doctoral School, “Victor Babes” University of Medicine and Pharmacy Timisoara, Eftimie Murgu Square 2, 300041 Timisoara, Romania; adelina.maritescu@umft.ro (A.M.); stoicescu.emil@umft.ro (E.R.S.); 2 Pulmonary Rehabilitation Center, Clinical Hospital of Infectious Diseases and Pulmonology, “Victor Babes”, Gheorghe Adam Street 13, 300310 Timisoara, Romania; pescaru.camelia@umft.ro; 3 Research Center for the Assessment of Human Motion, Functionality and Disability (CEMFD), “Victor Babes” University of Medicine and Pharmacy Timisoara, Eftimie Murgu Square 2, 300041 Timisoara, Romania; 4 Center of Research and Innovation in Personalized Medicine of Respiratory Disease (CRIPMRD), “Victor Babes” University of Medicine and Pharmacy Timisoara, Eftimie Murgui Square 2, 300041 Timisoara, Romania; oancea@umft.ro; 5 Faculty of Mechanics, Field of Applied Engineering Sciences, Specialization Statistical Methods and Techniques in Health and Clinical Research, “Politehnica” University Timisoara, Mihai Viteazu Boulevard No. 1, 300222 Timisoara, Romania; 6 Research Center for Pharmaco—Toxicological Evalutations, “Victor Babes” University of Medicine and Pharmacy Timisoara, Eftimie Murgu Square No. 2, 300041 Timisoara, Romania; iacob.daniela@umft.ro; 7 Pulmonology Clinic, Clinical Hospital of Infectious Diseases and Pulmonology, “Victor Babes”, Gheorghe Adam Street 13, 300310 Timisoara, Romania; 8 Departament of Neonatology, “Victor Babes” University of Medicine and Pharmacy Timisoara, Eftimie Murgu Square No. 2, 300041 Timisoara, Romania

**Keywords:** long COVID-19, pulmonary rehabilitation, progressive muscle relaxation, anxiety reduction, exercise capacity

## Abstract

**Background:** The aim of this study was to investigate the effects of pulmonary rehabilitation (PR) and additional progressive muscle relaxation (PMR) techniques in patients with long-term COVID-19 symptoms. **Methods**: We included 61 patients with long COVID-19 symptoms and randomly assigned them to two groups: PR only (group 1 with 30 subjects) and PR with PMR (group 2 with 31 subjects). The PR program consisted of gradual aerobic conditioning, strength training, and breathing exercises. Group 2 received additional 20 min daily sessions of progressive muscle relaxation techniques. **Results**: Following a 21-day intervention, it was observed that both groups had noteworthy improvements in lung function, exercise capacity, and sleep quality with statistical significance (*p* < 0.0001). Group 2 showed significant improvements in overall health (as measured by the General Health Questionnaire-12), patient health (as assessed by the Patient Health Questionnaire-9), general anxiety levels (as indicated by the Generalized Anxiety Disorders Scale-7), and sleep quality (as measured by the Pittsburgh Sleep Quality Index), with statistical significance (*p* < 0.0001), compared to group 1. Moreover, the statistical analysis demonstrated no significant difference in exercise capacity improvement between group 1 and group 2, as indicated by a *p*-value of 0.1711. **Conclusions:** The addition of progressive muscle relaxation to pulmonary rehabilitation significantly enhances mental health outcomes, particularly in reducing anxiety and improving sleep quality, for patients with long-term COVID-19 symptoms. These findings suggest that incorporating PMR into PR programs offers a valuable non-pharmacological approach to improving overall patient well-being during long-term COVID-19 recovery.

## 1. Introduction

COVID-19, an infectious disease caused by the SARS-CoV-2 virus, initially surfaced in Wuhan, China, in November 2019. Since then, it has swiftly disseminated globally, prompting the World Health Organization (WHO) to designate it as a pandemic in March 2020 due to its life-threatening nature [1].

Long-term COVID-19, also known as post-acute sequelae of SARS-CoV-2 infection, affects around 10–30% of those infected with COVID-19, with higher rates among those who experienced severe illness, such as hospitalization. The symptoms, which can persist for months, include fatigue, shortness of breath, brain fog, and muscle pain. The risk factors for long COVID include severe acute infection, older age, female gender, pre-existing conditions (e.g., diabetes, obesity, autoimmune diseases), and a higher number of initial symptoms. Vaccination significantly reduces the risk, with vaccinated individuals up to 50–70% less likely to experience long-term COVID-19. Early treatment and managing underlying health issues also lower the risk of prolonged symptoms [2].

Notably, COVID-19 patients faced a heightened risk of experiencing depression and anxiety [3]. Patients diagnosed with this disease necessitate isolation treatment, which has been observed to lead to anxiety and sleep disturbances in many cases [4].

Describing long-term COVID-19 as a condition and accurately assessing prevalence rates are crucial, yet neither task is simple. The difficulty in diagnosing based on symptom clusters arises from the prevalence of common long-term symptoms of COVID-19 (such as fatigue, headache, brain fog, musculoskeletal pain, shortness of breath, and chest pain) within the general population [5].

Anxiety, being a form of psychological stress, can initiate a cascade of physiological responses that ultimately result in a weakened immune system [6]. Professionals advise implementing interventions aimed at addressing anxiety, depression, sleep issues, and symptoms of post-traumatic stress disorder, either directly or indirectly, to enhance the mental well-being of this population [7]. To address anxiety, a combination of pharmacological and non-pharmacological approaches can be employed. While medications like benzodiazepines may provide initial relief for anxiety and sleep issues in post-COVID-19 patients, they are not sustainable long-term solutions [8]. Alternatively, non-pharmacological methods such as pulmonary rehabilitation (PR) and progressive muscle relaxation (PMR) offer effective means of managing anxiety and sleep disorders in these patients [9,10,11].

Pulmonary rehabilitation is vital for post-COVID-19 patients. It provides exercises and therapies to restore lung function, strengthen respiratory muscles, and improve quality of life [12,13]. It also addresses mental health, educates patients about medication use, and empowers them with self-management knowledge. Regular assessments and professional guidance ensure personalized care and improve the overall quality of life by addressing recovery’s physical and psychological aspects [13,14,15].

Progressive muscle relaxation is a technique that does not necessitate specialized equipment, specific settings, or a fixed treatment duration [16]. Numerous studies have demonstrated the efficacy of PMR exercises in ameliorating sleep quality and reducing anxiety levels [17,18,19,20]. While previous studies have shown the effectiveness of PMR in improving sleep quality and reducing anxiety levels in patients with COPD, there is limited research on the application of PMR in patients with long-term symptoms of COVID-19.

This study’s objective was to evaluate and compare the effectiveness of pulmonary rehabilitation alone and in combination with progressive muscular relaxation in improving physical function, mental health, and sleep quality in patients experiencing long-term symptoms of COVID-19. Specifically, thus study aimed to determine if the addition of PMR to PR can provide superior benefits in alleviating anxiety, depression, and sleep disturbances compared to PR alone.

## 2. Materials and Methods

This study followed the CONSORT guidelines for reporting randomized controlled trials to ensure transparency and rigor in reporting the trial’s methodology and findings [21].

### 2.1. Study Design and Randomization

Participants were randomized using MeldCalc with a block size of 4, ensuring balanced allocation to the PR group or the PR-combined-with-progressive muscle relaxation group by pre-randomizing block sequences and assigning participants based on these sequences as they enrolled. We used a single-masked design where the participants were unaware of whether they were assigned to the PR group or the PR-combined-with-PMR group. At the same time, the outcome assessor remained blinded to the group assignments to minimize performance and assessment bias. This research study gained approval from the ethics committee of the Victor Babes Clinical Hospital (4990) and was registered on clinicaltrials.gov (NCT06492577), according to the requirements for reporting randomized controlled trials.

Participants were included in the study if they met the following criteria: a confirmed diagnosis of COVID-19 through a documented positive RT-qPCR or antibody test; persistent long COVID-19 symptoms, specifically moderate to severe dyspnea and fatigue, lasting at least three months post-infection; an age between 18 and 75 years; a stable medical condition; no recent exacerbations or hospital admissions for other conditions in the past three months.

Participants were excluded from the study if they had severe comorbid conditions, such as moderate to severe heart disease, severe ischemic or hemorrhagic stroke, neurodegenerative diseases, or severe acute illnesses; if they had undergone major surgery or been hospitalized for severe conditions within the past six months; if they had severe cognitive or psychiatric disorders; if they had active respiratory infections; if they were immunocompromised; if they had severe mobility impairments or chronic pain that could limit their participation in rehabilitation exercises; or if they engaged in high alcohol intake or substance abuse.

**Figure 1:**
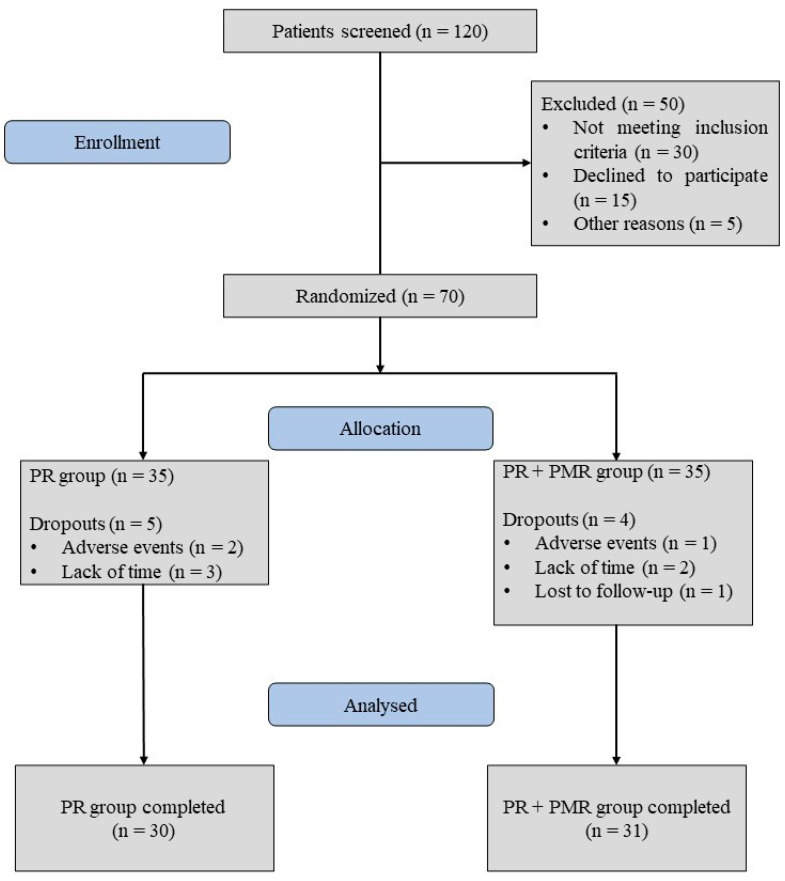
Flowchart of inclusion and exclusion.

We included 61 post-COVID-19 patients who came to the hospital for pulmonary rehabilitation. The pulmonary rehabilitation program lasted for 21 days. A flowchart provides a detailed illustration of the process of patient screening, inclusion, randomization, and the follow-up of the study (Figure 1).

### 2.2. Pulmonary Function

We evaluated pulmonary function using spirometry, where we measured forced vital capacity (FVC) and forced expiratory volume in one second (FEV_1_) and modified the Tiffeneau–Pinelli index (FEV_1_/FVC). COSMED Quark PFT equipment was used to perform the lung function tests. Spirometry was performed using appropriate techniques, following the American Thoracic Society and the European Respiratory Society (ATS-ERS) guidelines [22].

### 2.3. General Health

Participants’ psychological well-being was assessed using the GHQ-12. This questionnaire comprises 12 questions, which patients rate on a four-point Likert scale. The GHQ-12 is a valuable tool for identifying specific psychological factors. This study focused on assessing the following items: anxiety and depression, social dysfunction, and loss of confidence. Each of these factors is associated with specific items of the GHQ-12, and their assessment is determined by the cumulative scores obtained from the questionnaire items [23].

### 2.4. Patient Health

The Patient Health Questionnaire-9 (PHQ-9), a component of the Comprehensive Patient Health Questionnaire (PHQ), is a self-administered instrument used to diagnose major and subthreshold depression in the general population. This scale assesses the intensity of symptoms experienced in the last two weeks, and the answers are scored on a scale of 0 to 3. The total score can vary between 0 and 27. The scale indicate an absence or minimal depressive symptoms at a score between 0 and 4; mild depression at a score between 5 and 9; more pronounced symptoms, i.e., moderate depression, at a score between 10 and 14; significant symptoms that affect the patient’s daily life, where clinical intervention is necessary, when the score is between 15 and 19; and severe depression when the score is between 20 and 27 [24].

### 2.5. General Anxiety

The Generalized Anxiety Disorder Scale (GAD-7) serves as a valuable self-report anxiety measurement tool. It gauges the intensity of symptoms experienced in the past two weeks. Each of its seven items is rated on a four-point severity scale (0 = not at all, 1 = a few days, 2 = more than half of the days, 3 = almost every day). The total scores on this scale span from 0 to 21. Higher scores indicate more pronounced anxiety symptoms. A score of 10 or higher signifies moderate to severe anxiety symptoms, potentially marking a clinically significant condition [24].

### 2.6. Sleep Quality

The PSQI (Pittsburgh Sleep Quality Index) assesses sleep quality and related issues over one month. This questionnaire comprises nineteen self-report questions categorized into seven components: 1. sleep quality, 2. sleep latency, 3. sleep duration, 4. sleep efficiency, 5. sleep disorders, 6. use of sleep medications, and 7. daytime sleepiness. Each of these components is assigned a score ranging from 0 to 3. The scores vary from 0 to 21, where a PSQI score equal to or lower than 5 indicates good sleep quality, while a score higher than 5 suggests poor sleep quality [25].

### 2.7. Exercise Capacity

We used the 6 min walking test (6MWT) to assess exercise capacity. This assessment involves the patient attempting to walk the furthest possible distance within a specified time frame. To perform the 6MWT, we used the ERS/ATS record form, the BORG scale, a pulse oximeter, and a stopwatch. Assessments were performed according to the ATS guidelines. The main outcome is the distance covered, with healthy adults usually covering more than 80% of the predicted distance calculated according to age, height, and weight, which indicates an average exercise capacity [26].

### 2.8. Intervention

#### 2.8.1. Pulmonary Rehabilitation Program

The pulmonary rehabilitation program was structured according to the American Thoracic Society (ATS) guidelines [27] and designed using the FITT (Frequency, Intensity, Time, and Type) principle. The program aimed to improve lung function, exercise capacity, and overall physical health in patients with long-term COVID-19 symptoms. The PR program lasted 21 days, progressively increasing exercise intensity and duration based on each patient’s abilities and tolerance. The details of the program are provided in Table 1.

#### 2.8.2. Progressive Muscle Relaxation Program

The progressive muscle relaxation program was designed to complement the pulmonary rehabilitation program by reducing anxiety and improving sleep quality in patients with long-term COVID-19 symptoms. The PMR program lasted 21 days, with daily PMR sessions guided by a structured program. The details of the program are provided in Table 2.

The daily PMR session structure provided in Table 3 details the specific components of each PMR session to ensure consistent practice and maximize the benefits.

### 2.9. Statistical Analysis

All data and analyses were processed with a licensed version of MedCalc^®^ Statistical Software version 20.118 (MedCalc Software Ltd., Ostend, Belgium; https://www.medcalc.org; 2023).

Given these parameters (GHQ-12, PHQ-9, GAD-7, PSQI) post-PR and post-PR and -PMR, a sample size calculator for an independent samples *t*-test indicates that a minimum sample size of approximately 12 per group would be required to detect a significant difference (>3 units) between the means with the provided effect size, assuming a desired power of 0.80 and a significance level of 0.05. For a desired power of 0.99 and a significance level of 0.01, a minimum sample size of approximately 26 per group would be necessary.

### 2.10. Participants

We randomly assigned the 61 patients to two groups using the an online randomization tool. Upon enrollment, patients were allocated to the PR program, with the others receiving PMR as an additional intervention. The first group contained 30 patients, while the second had 31 patients. The parameters age, gender, weight, height, and BMI were noted as Group 1 or Group 2, depending the each group.

The attrition rate was calculated based on the total number of participants at the start of the study and the number of participants who dropped out or did not complete the intervention. In this study, of the total of 61 patients initially enrolled, 30 were assigned to the pulmonary rehabilitation (PR) program, and 31 received progressive muscle relaxation (PMR) as an additional intervention.

The plot distribution was analyzed by conducting a Shapiro–Wilk test. According to these results, the parameters age, FVC, FEV_1_, GHQ-12, PHQ-9, GAD-7, and PSQI showed a significant departure from normality, which required non-parametric tests. The Shapiro–Wilk test demonstrated a parametric distribution for the rest of the parameters. The central tendency indicators were described as the arithmetic mean and dispersion—standard deviation (S.D.) for parametric variables and medians and interquartile range [IQR] for non-parametric ones. The parameters (continuous variables) were compared using an independent samples *t*-test or Mann–Whitney test (independent samples). A *p*-value *p* < 0.05 was considered significant.

## 3. Results

### 3.1. Baseline Characteristics of Patients

Of the total of 30 subjects from group 1, 22 (73.33%) were male. The age range of patients was 54–74, with a median age of 66.50 [63.00; 70.00], presented as median and [IQR]. The height of the patients varied d 183 cm, and the mean was 170.63 ± 6.78 (arithmetic mean ± S.D.). The patients’ weight varied between 55 and 94 kg, with a mean of 75.16 ± 10.36. The patients’ BMI ranged from 18.7 to 31.9; 25.80 ± 3.38.

Of the total of 31 subjects from group 2, 22 (74.19%) were male. The age range of patients was 56–72, with a median age of 64; [62.00; 68.00], presented as median and [IQR]. The height of the patients varied between 159 and 183 cm, and the mean was 173.06 ± 5.83 (arithmetic mean ± S.D.). The patients’ weight varied between 64 and 98 kg, with a mean of 77.97 ± 9.68. The BMI of the patients ranged between 21.4 and 31.6; 25.90 ± 2.48. The patients’ characteristics are presented in Table 4.

### 3.2. Parameter Analysis for Group 1 and Group 2 Pre- and Post-PR Program

The variation in patients’ parameters before and after the PR program and PR-and-PMR program are presented in Table 5 below.

### 3.3. Comparison of Analyzed Parameters for Groups

**Figure 2:**
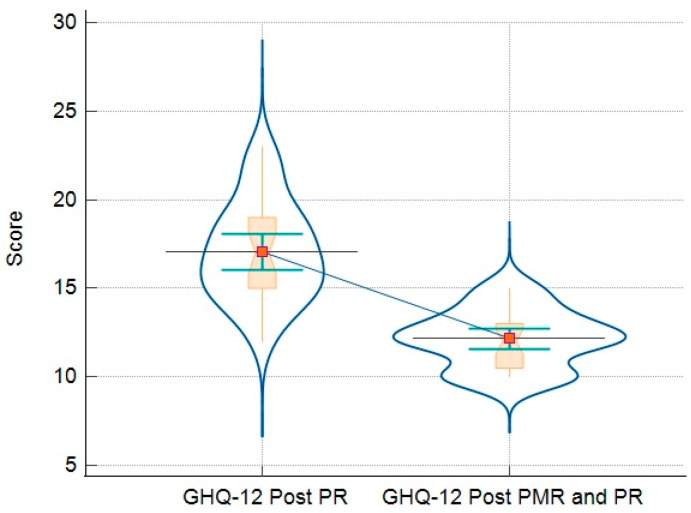
Relationship between GHQ-12 results of patients post-PR (left side) and post-PR-and-PMR (right side)—dot-and-line diagram (violin representation with connecting lines, markers, and dots that plot all data).

**Figure 3:**
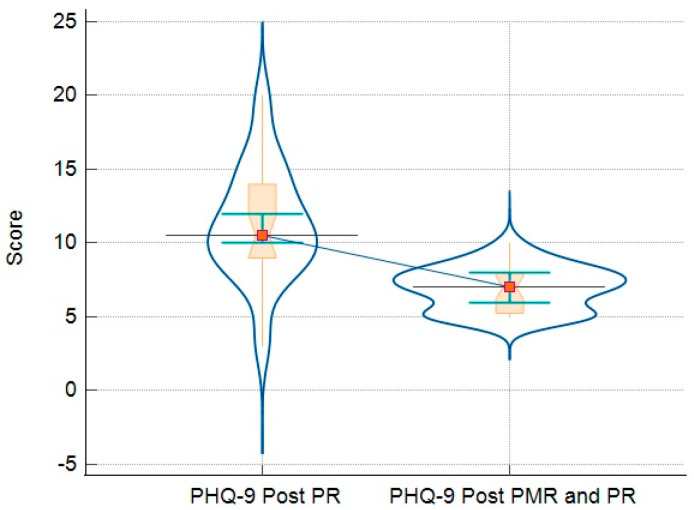
Relationship between PHQ-9 results of patients post-PR (left side) and post-PR-and-PMR (right side)—notched box-and-whisker plot with connecting lines, markers, and bars.

**Figure 4:**
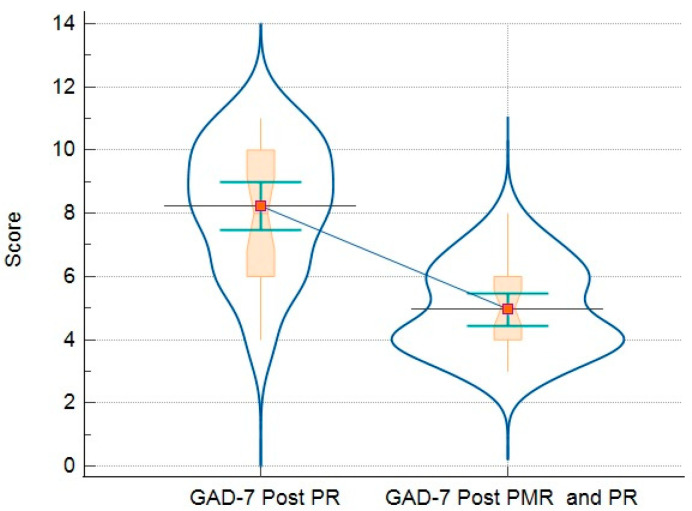
Relationship between GAD-7 results of patients post-PR (left side) and post-PR-and-PMR (right side)—box-and-whisker plot with connecting lines, markers, and intervals.

**Figure 5:**
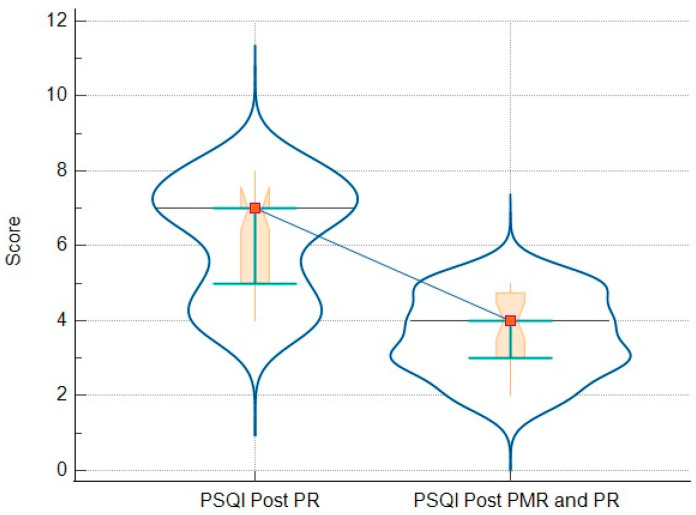
Relationship between PSQI results of patients post-PR (left side) and post-PR-and-PMR (right side)—box-and-whisker plot with connecting lines, markers, and intervals.

The final analysis compares the results of the questionnaires and objective measures (such as 6MWT) in patients who only followed PR vs. patients who followed the PR-and-PMR program. These results are detailed in Table 6. In addition, there are graphic representations that demonstrate all the variations in GHQ-12 (Figure 2), PHQ-9 (Figure 3), GAD-7 (Figure 4), and PSQI (Figure 5).

## 4. Discussion

To our knowledge, this is the first study that evaluates the combination of PR and PMR for long-term COVID-19 patients, focusing on mental health outcomes. We observed that PR alone and PR combined with PMR led to significant improvements in exercise capacity, evaluated by the 6MWT, and sleep quality, assessed with the PSQI. In Group 1, the median FVC% increased from 71.50% to 76% post-intervention. While this increase was statistically significant (*p* < 0.001), we observed that the improvement in functional status, as measured by the 6 min walking test (6MWT), was more modest. The mean 6MWT distance in Group 1 increased from 332.06 m to 366.60 m, representing a functional improvement but not one that was necessarily proportionate to the change in FVC%. This suggests that while FVC% improved, the translation to functional gains, particularly in exercise capacity, may have been limited.

However, the group that received the addition of PMR experienced more substantial benefits in terms of mental health outcomes. Improvements in psychological well-being were observed, with the GHQ-12 scores reduced by a mean of 4.9 points. In studies that use the Likert scale to calculate the GHQ-12 score, cut-off points can vary, but scores above 12–15 are often considered indicative of mental distress [28]. Participants in the group receiving PR and PMR had a significantly lower GHQ-12 score of 12.16, reflecting a more significant improvement.

Regarding the measures of depression screening, both groups were at the threshold of moderate depressive symptoms, evaluated with the PHQ-9, at the beginning of the study. A 5-point change in the scale is considered clinically significant [29]. We observed that the intervention group had a substantial reduction in depressive symptoms, reaching the threshold of mild depressive symptoms. Regarding the GAD-7 questionnaire, we observed that both groups reached the lower–moderate generalized anxiety threshold limit. Both groups experienced a reduction in anxiety scores, but the intervention group reached the lower threshold of mild anxiety. Moreover, patients performing PMR had a decrease of 4.9 points in GAD-7, where the estimated MCID is 4 points [30], meaning that this intervention can significantly reduce the symptoms of anxiety.

While pulmonary rehabilitation alone leads to some improvement in sleep quality, we observed that the addition of PMR provided a significant and clinically relevant enhancement in sleep quality, as measured with the PSQI. The MCID for the PSQI is typically considered a reduction of three points from the baseline value [31]. We observed that the PMR group’s PSQI scores were reduced by 4.74 points. Moreover, this group went from a median score of 8.35, indicating poor sleep quality, to a mean score of 4.96, indicating no more poor sleep quality.

Seyedi et al. showed that PMR can relieve fatigue and enhance sleep quality in patients with COPD [32]. The reason for the decrease in patients’ anxiety after PMR training may be the balance between the anterior nucellus and the hypothalamus. By reducing the activity of the sympathetic nervous system, the side effects of stress and anxiety can be prevented, and physical and mental relaxation can be increased [33].

This study showed no significant changes in functional capacity in both groups. However, compared to PR alone, this combination significantly improved sleep quality and decreased fatigue and anxiety in patients with COVID-19. Despite the previously reported adverse effects of fatigue on functional capacity [34,35,36], the present study did not show differences in functional capacity between the two groups. Our findings are consistent with the results obtained by Cortes-Telles et al., who reported insignificant differences in pulmonary function and 6MWT scores between patients with COVID-19 with and without fatigue [37].

Although the etiology remains unclear, fatigue is known to be complicated and multifactorial in terms of its mechanism [38]. No distinct immunological findings associated with fatigue have been observed in patients with COVID-19. Therefore, non-pharmacological interventions are recommended to treat fatigue in these patients [39]. Kentson et al. showed that fatigue has a more significant relationship with psychological factors than physiological factors in COPD [40]. Considering that stress is a reported risk factor for fatigue after acute infections [41] and that fatigue is correlated with sleep quality [42], we can assume that PMR can reduce fatigue and trigger psychophysical relaxation by decreasing the sympathetic nervous system’s activity and preventing the side effects of stress and anxiety [43]. Park et al. found that PMR reduced heart and respiratory rate, systolic and diastolic blood pressure, and salivary cortisol levels, which could explain the effect of PMR on anxiety in this study [44]. These findings are consistent with those reported by Liu et al. [6], and Xiao et al. [10] found that PMR performed for 5 and 7 days reduced anxiety and improved sleep quality compared to routine care in patients with COVID-19.

The anxiety levels of the study group decreased significantly compared to the control group. Progressive muscle relaxation techniques can effectively reduce the anxiety of patients with COVID-19 regarding difficult situations, such as hospitalization, treatment, and isolation [6]. Liu et al. administered PMR exercises to patients with COVID-19 for 30 min a day for five days and found that the techniques effectively reduced their anxiety levels [6]. Manzoni et al. conducted a 10-year systematic review with a meta-analysis, and discovered that muscle relaxation training is consistently practical and should be considered for reducing anxiety [45]. Volapto et al. performed a meta-analysis of 25 randomized controlled trials of muscle relaxation exercises in patients with COPD and found that the practices significantly reduced anxiety [46].

The sleep quality in the patients in the group that performed PMR increased significantly compared to the group that only performed PR. Our findings suggest that PMR exercises effectively eliminate sleep problems in patients with COVID-19. This result is similar to that of the study by Ibrahim Ozlu et al., in which they found that PMR also improved sleep quality in patients with COVID-19 [47]. Yilmaz et al. systematically reviewed muscle relaxation exercises in COPD patients and found that the exercises effectively reduce fatigue, anxiety, and depression and improve sleep quality [48]. Aksu et al. found that PMR exercises prevent the deterioration of sleep quality in patients with pulmonary resection [17]. Chegeni et al. conducted a randomized controlled trial with COPD patients and reported that PMR exercises effectively improve subjective sleep quality [32].

The results of this study are similar to those of studies conducted on various patient groups that have shown that PMR exercises effectively prevent sleep problems. Studies on multiple sclerosis, hemodialysis, cancer [49], and intensive care patients [50] determined that PMR exercises significantly improve sleep quality.

Adding progressive muscle relaxation techniques to the pulmonary rehabilitation program offers a non-pharmacological approach to alleviate anxiety and enhance the quality of sleep in patients with long-term COVID-19 symptoms. PMR is advantageous because medications prescribed for anxiety and sleep issues in COVID-19 patients often come with various side effects that may hinder monitoring signs.

One limitation of this research study is the limited generalizability of the findings. The study population consists of only 61 post-COVID-19 patients who came to the hospital for pulmonary rehabilitation, which may not represent the broader population of individuals experiencing long-term COVID-19 symptoms. In our study, the participants’ ages ranged from 54 to 74 years, with a median age of 66.50 years in Group 1 (PR only) and 64 years in Group 2 (PR + PMR). While long COVID-19 can affect individuals across a wide age range, the inclusion criteria for this study focused on older adults, as they tend to experience more severe long-term symptoms. The strict inclusion and exclusion criteria further restrict the diversity of the sample, potentially excluding individuals with different characteristics or comorbidities that could affect the outcomes. Therefore, this study’s findings may not apply to a more diverse and heterogeneous population of long COVID-19 patients.

Another limitation of this study is the relatively short duration of the pulmonary rehabilitation program, which lasted for 21 days. Long COVID-19 symptoms can persist for an extended period, often months or years. A 21-day program may not provide a sufficient timeframe to observe the full extent of improvement in symptoms, especially considering the complex and chronic nature of conditions like anxiety, fatigue, and sleep disturbances. Longer-term follow-up assessments would be necessary to assess the sustainability of any improvements observed during the intervention and to capture potential changes in symptoms over time.

## 5. Conclusions

Combining pulmonary rehabilitation with progressive muscle relaxation significantly enhances mental health outcomes, particularly by reducing anxiety and improving sleep quality, in patients with long-term COVID-19 symptoms. These findings suggest that integrating PMR into PR programs offers a valuable, non-pharmacological approach to comprehensive patient care during long-term COVID-19 recovery.

## Figures and Tables

**Table 1:** Pulmonary rehabilitation program protocol.

Phase	Days	Exercise Type	Frequency	Intensity	Duration	Details
Initial phase	1–7	Aerobic exercise	5 days/week	Moderate (4–6 on Borg RPE scale)	20–30 min	Walking, cycling
		Strength training	3 days/week (non-consecutive)	50% of 1RM, 1–2 sets of 8–10 reps	20–30 min	Body weight, resistance bands
		Breathing exercises	Daily	Controlled breathing	10 min	Diaphragmatic, pursed-lip breathing
Progressive phase	8–14	Aerobic exercise	5 days/week	Moderate (4–6 on Borg RPE scale)	30–40 min	Brisk walking, moderate cycling
		Strength training	3 days/week (non-consecutive)	60% of 1RM, 2–3 sets of 10–12 reps	20–30 min	Increasing resistance as tolerated
		Breathing exercises	Daily	Controlled breathing	10–15 min	Continued practice, lung expansion techniques
Advanced phase	15–21	Aerobic exercise	5 days/week	Moderate (4–6 on Borg RPE scale)	40–45 min	Vigorous cycling, treadmill exercises
		Strength training	3 days/week (non-consecutive)	70% of 1RM, 2–3 sets of 12 reps	20–30 min	Progressive increase in resistance
		Breathing exercises	Daily	Controlled breathing	10–15 min	Advanced techniques for maximizing lung capacity and efficiency

**Table 2:** Progressive muscle relaxation program protocol.

Phase	Days	Focus	Session Duration	Details
Initial phase	1–7	Introduction to PMR techniques	20 min daily	Day 1–3: Lower body (feet, calves, thighs)
				Day 4–5: Middle body (abdomen, lower back, chest)
				Day 6–7: Upper body (hands, arms, shoulders)
Progressive Phase	8–14	Enhancing relaxation and breathing techniques	20 min daily	Day 8–10: Full body PMR from feet to head
				Day 11–12: Emphasis on high-tension areas (shoulders, neck)
				Day 13–14: Reinforcement of relaxation techniques with deep breathing
Advanced Phase	15–21	Maximizing relaxation and visualization	20 min daily	Day 15–17: Full body PMR with enhanced deep breathing
				Day 18–19: Incorporation of visualization techniques
				Day 20–21: Consolidation of techniques, focus on sustained relaxation and stress management

**Table 3:** Daily PMR structure.

Component	Duration	Details
Preparation	2 min	In a quiet, comfortable place; sitting or lying in a relaxed position; eyes closed, taking a few slow, deep breaths.
Muscle Tensing and Relaxation	15 min	Tensing each muscle group for 5 s, then relaxing for 20 s. Following this sequence: feet, calves, thighs, abdomen, lower back, chest, hands, arms, shoulders, neck, face (jaw, eyes, forehead).
Cool Down	3 min	Continuing slow, deep breathing; focusing on the feeling of relaxation.

**Table 4:** A comparison of the baseline characteristics between the two groups.

Parameters	Group 1	Group 2	*p*-Value
Male gender	22 (73.33%)	22 (74.19%)	0.93
Age	66.50; [63.00; 70.00]	64; [62.00; 68.00]	0.12
Height	170.63 ± 6.78	173.06 ± 5.83	0.13
Weight	75.16 ± 10.36	77.97 ± 9.68	0.27
BMI	25.80 ± 3.38	25.90 ± 2.48	0.89
FVC (L)	2.81; [2.62; 3.33]	2.75; [2.62; 3.04]	0.74
FVC (%)	71.50; [67; 81]	72; [67; 78]	0.78
FEV_1_ (L)	2.31; [2.15; 2.78]	2.32; [2.18; 2.60]	0.97
FEV_1_ (%)	78; [66; 89]	75; [64; 86.75]	0.53
FEV_1_/FVC	84; [75; 86]	85; [76; 88.75]	0.28
6MWT (m)	332.06 ± 86.28	347.25 ± 61.94	0.43
6MWT (%)	71.50 ± 15.86	73.38 ± 12.81	0.61
GHQ-12	20; [18; 21]	20; [20; 22.75]	0.28
PHQ-9	13.60 ± 3.23	13.48 ± 3.26	0.88
GAD-7	10; [8; 11]	10; [8; 11]	0.98
PSQI	8; [6; 9]	8; [8; 9]	0.07

Data are expressed as arithmetic mean ± S.D./median and [IQR] using independent samples *t*-test and Mann—Whitney U test. BMI—body mass index; FVC—forced vital capacity; FEV_1_—forced expiratory volume in the first second; 6MWT—six-minute walking test; GHQ-12—General Health Questionnaire with 12 items; PHQ-9—Patient Health Questionnaire with 9 items; GAD-7—General Anxiety Disorder with 7 items; PSQI—Pittsburgh Sleep Quality Index.

**Table 5:** Variation in patients’ parameters before and after intervention for both.

Patients’ Parameters	Pre-PR Program Group 1	Post-PR Program Group 1	*p*-Value	Pre-PR-and-PMR Program Group 2	Post-PR-and-PMR Program Group 2	*p*-Value
FVC (L)	2.81; [2.62; 3.33]	3.01; [2.80; 3.53]	<0.001	2.75; [2.62; 3.04]	3.15; [3.00; 3.48]	0.001
FVC (%)	71.50; [67; 81]	76; [71; 85]	<0.001	72; [67; 78]	76; [69.25; 83]	0.198
FEV (L)	2.31; [2.15; 2.78]	2.50; [2.32; 2.99]	<0.001	2.32; [2.18; 2.60]	2.55; [2.41; 2.85]	<0.001
FEV (%)	78; [66; 89]	84.50; [73; 94]	<0.001	75; [64; 86.75]	79; [71.75; 94.50]	<0.001
FEV_1_/FVC	84; [75; 86]	85.50; [76; 88]	0.001	85; [76; 88.75]	85; [75; 92.75]	0.922
6MWT (m)	332.06 ± 86.28	366.60 ± 81.97	<0.001	347.25 ± 61.94	391.54 ± 56.81	<0.001
6MWT (%)	71.50 ± 15.86	79.03 ± 14.81	<0.001	73.38 ± 12.81	82.77 ± 11.35	<0.001
GHQ-12	20; [18; 21]	17; [15; 19]	<0.001	20; [20; 22.75]	12; [10.50; 13]	<0.001
PHQ-9	14; [11; 16]	10.50; [9; 14]	<0.001	13; [10.25; 15.75]	7; [5.25; 8]	<0.001
GAD-7	10; [8; 11]	8; [6; 10]	<0.001	10; [8; 11]	5; [4; 6]	<0.001
PSQI	8; [6; 9]	7; [5; 7]	<0.001	8; [8; 9]	4; [3; 4.75]	<0.001

Data are expressed as arithmetic mean ± S.D./median and [IQR] using Wilcoxon signed-rank test and paired samples *t*-test. FVC—forced vital capacity; FEV_1_—forced expiratory volume in the first second; 6MWT—six-minute walking test; GHQ-12—General Health Questionnaire with 12 items; PHQ-9—Patient Health Questionnaire with 9 items; GAD-7—General Anxiety Disorder with 7 items; PSQI—Pittsburgh Sleep Quality Index.

**Table 6:** Comparison of patients’ analyzed parameters post-PR for group 1 and post-PR-and-PMR for group 2.

Patients’ Parameters	Post-PR Group 1	Post-PR-and-PMR Group 2	*p*-Value
GHQ-12	17.06 ± 2.76	12.16 ± 1.59	<0.001
PHQ-9	10.50; [9; 14]	7; [5.25; 8]	<0.001
GAD-7	8.23 ± 2.02	4.96 ± 1.40	<0.001
PSQI	7; [5; 7]	4; [3; 4.75]	<0.001
6MWT	366.60 ± 81.97	391.54 ± 56.81	0.171
6MWT (%)	79.03 ± 14.81	82.77 ± 11.35	0.271

Data are expressed as arithmetic mean ± S.D. or median and [IQR] using independent samples *t*-test and Mann–Whitney U test. GHQ-12—General Health Questionnaire; PHQ-9—Patient Health Questionnaire; PSQI—Pittsburgh Sleep Quality Index; 6MWT—six-minute walking test.

## Data Availability

The supporting data for the findings of this study can be obtained by contacting the corresponding author. However, the data cannot be publicly accessed due to privacy and ethical considerations.
